# AdjustSense: Adaptive 3D Sensing System with Adjustable Spatio-Temporal Resolution and Measurement Range Using High-Speed Omnidirectional Camera and Direct Drive Motor

**DOI:** 10.3390/s21216975

**Published:** 2021-10-21

**Authors:** Mikihiro Ikura, Sarthak Pathak, Jun Younes Louhi Kasahara, Atsushi Yamashita, Hajime Asama

**Affiliations:** 1 Department of Precision Engineering, The University of Tokyo, Hongo, Bunkyo-ku, Tokyo 113-8656, Japan; louhi@robot.t.u-tokyo.ac.jp (J.Y.L.K.); yamashita@robot.t.u-tokyo.ac.jp (A.Y.); asama@robot.t.u-tokyo.ac.jp (H.A.); 2 Department of Precision Mechanics, Faculty of Science and Engineering, Chuo University, Kasuga, Bunkyo-ku, Tokyo 112-8551, Japan; pathak@mech.chuo-u.ac.jp

**Keywords:** 360^∘^ 3D sensing, local 3D sensing, spatio-temporal resolution, measurement range, light-section method

## Abstract

Many types of 3D sensing devices are commercially available and were utilized in various technical fields. In most conventional systems with a 3D sensing device, the spatio-temporal resolution and the measurement range are constant during operation. Consequently, it is necessary to select an appropriate sensing system according to the measurement task. Moreover, such conventional systems have difficulties dealing with several measurement targets simultaneously due to the aforementioned constants. This issue can hardly be solved by integrating several individual sensing systems into one. Here, we propose a single 3D sensing system that adaptively adjusts the spatio-temporal resolution and the measurement range to switch between multiple measurement tasks. We named the proposed adaptive 3D sensing system “AdjustSense.” In AdjustSense, as a means for the adaptive adjustment of the spatio-temporal resolution and measurement range, we aimed to achieve low-latency visual feedback for the adjustment by integrating not only a high-speed camera, which is a high-speed sensor, but also a direct drive motor, which is a high-speed actuator. This low-latency visual feedback can enable a large range of 3D sensing tasks simultaneously. We demonstrated the behavior of AdjustSense when the positions of the measured targets in the surroundings were changed. Furthermore, we quantitatively evaluated the spatio-temporal resolution and measurement range from the 3D points obtained. Through two experiments, we showed that AdjustSense could realize multiple measurement tasks: 360${}^{\circ }$ 3D sensing, 3D sensing at a high spatial resolution around multiple targets, and local 3D sensing at a high spatio-temporal resolution around a single object.

## 1. Introduction

3D sensing is a critical technology for machines that allows them to recognize spatial expanse and temporal changes in the environment. Moreover, 3D sensing technology is utilized in many technical fields, such as robotics and infrastructure [1,2,3,4,5]. Effective and efficient methods for 3D sensing were proposed for application in these fields [6,7,8]. Recently, many 3D sensors incorporating these methods became commercially available, allowing people to use 3D sensing technologies easily [9,10].

Most 3D sensors have a constant spatio-temporal resolution and measurement range, providing the user with steady measurement results. The spatial resolution indicates the density of the measured point cloud, the temporal resolution indicates the frequency of measurement updates, and the measurement range indicates the angle range of horizontal and vertical scanning within which it is possible to measure 3D points. These constant specifications of the 3D sensing systems and sensors are determined according to measurement targets; hence, 3D sensing systems have specific strengths and weaknesses that depend on the measurement targets. For example, an omnidirectional LIDAR has the advantage that it can obtain a 360${}^{\circ }$ 3D sensing result over a large distance range all at once. However, LIDAR has difficulties capturing small fluctuations in the pose of the measurement target owing to the low temporal resolution of the 3D sensing. Moreover, LIDAR has low spatial resolution in the direction perpendicular to the rotation, causing difficulties for detail shape recognition. On the other hand, some 3D sensing systems based on the structured light method using cameras exhibit higher spatio-temporal resolution at the local measurement range [11,12,13], realizing not only detail shape recognition but also tracking fast moving measurement targets. However, the measurement range in which the structured light can be irradiated without blurs is limited. Consequently, this limitation in the measurement range makes it difficult to measure multiple targets simultaneously at high spatio-temporal resolution. To compensate for these disadvantages of 3D sensing systems and work with a variety of measurement targets simultaneously, it is essential to integrate multiple 3D sensing systems into one. However, the integration requires spatial arrangement and this requirement restricts the number of sensors and devices for 3D sensing that can be installed, depending on the system. Therefore, the objective of this study is to realize a single 3D sensing system that can switch between multiple measurement tasks instead of using multiple sensors and devices for 3D sensing.

**Figure 1:**
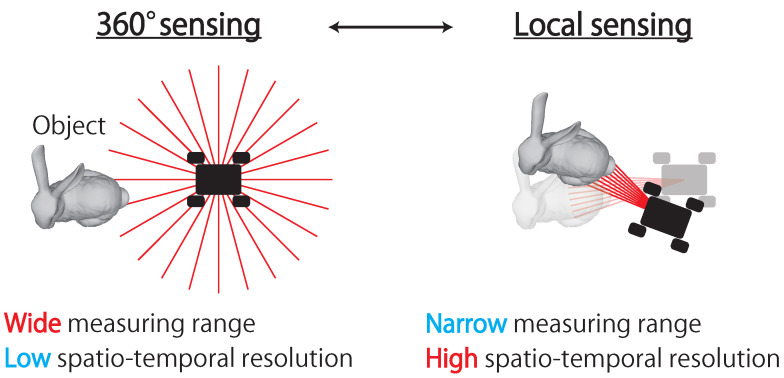
Mission scenario with adaptive 3D sensing system, which is referred to as “AdjustSense”.

Considering this objective, we propose an adaptive 3D sensing system that measures not only the 360${}^{\circ }$ circumference, but also a local area at a high spatio-temporal resolution as shown in Figure 1. The proposed adaptive 3D sensing system is referred to as “AdjustSense” in this study. As a means for the adaptive adjustment of the spatio-temporal resolution and measurement range, AdjustSense aims to achieve low-latency visual feedback by integrating a high-speed sensor and a high-speed actuator. The low-latency visual feedback realizes multiple measurement tasks simultaneously according to the surrounding environment with only a single measurement system. An example of a task scenario using a mobile robot equipped with AdjustSense is shown in Figure 1. AdjustSense scans the surroundings to detect objects approaching from arbitrary positions. If an object is detected as a measurement target by the 360${}^{\circ }$ 3D sensing, AdjustSense focuses the measurement range from 360${}^{\circ }$ to the periphery of the detected object. This local 3D sensing enables the measurement of an object at a high spatio-temporal resolution for this focused measurement range. Moreover, even if the measured object or the mobile robot moves, AdjustSense adaptively controls the direction of the line laser with low latency to continue measuring the object.

## 2. Related Work

### 2.1. High-Speed 3D Sensing

Recently, high-speed 3D sensing systems using high-speed cameras were developed [12,13,14,15]. Tabata et al. proposed a real-time 3D sensing system at 500 fps using two high-speed cameras and a high-speed projector [14]. In the system, the projector irradiates a segmented pattern as a structured light, and two synchronized high-speed cameras obtain images of the irradiated segmented patterns. By using the two images obtained at 500 fps and the epipolar constraints, numerous 3D points of an object can be measured. Moreover, Namiki et al. proposed an active vision system using a high-speed camera at 500 fps together with a high-speed projector attached to a two-axis pan-tilt actuator [12]. This system can not only measure the 3D points of a target object, but also track the object by controlling the two-axis actuator at a high speed.

However, although these 3D sensing systems can measure the shape of an object at high speeds, the spatial resolutions of the 3D sensing systems are constant, depending on the projected structured light. Moreover, Tabata’s 3D sensing system is limited in that the measurement range is only within the area of the projected structured light whose relative position to the two high-speed cameras is fixed. Compared with Tabata’s 3D sensing system, Namiki’s system expands the measurement range using a two-axis actuator. However, it is difficult to keep changing the measurement range at high speed because of the large inertia of the high-speed camera and high-speed projector attached to the actuator.

To realize an adaptive change in the spatio-temporal resolution and the measurement range with low latency, we propose a 3D sensing system that combines high-speed sensing with high-speed actuation. In the proposed 3D sensing system, a direct drive motor can control the direction of the line laser at high speed because the inertia of the line laser is small. According to the movement of the motor, the spatio-temporal resolution and measurement range can be adjusted. Furthermore, a high-speed camera can obtain images without motion blur of the line laser even when the line laser moves quickly; the 3D positions of the bright points of the line laser can be calculated based on the light-section method.

### 2.2. Adaptive 3D Sensing

Regarding conventional 3D sensing systems, the spatio-temporal resolution and measurement range are constant for each system. In contrast, Nagai et al. proposed an adaptive 3D sensing system in which the trade-off relationship between the spatial and temporal resolutions can be adaptively controlled [16]. In Nagai’s 3D sensing system, a 3D LIDAR with a wide field-of-view angle is attached to a rotating device, and the spatio-temporal resolution of the 3D sensing system is adaptively changed by controlling the rotation speed. Firstly, Nagai’s 3D sensing system evaluates the spatial unevenness of the surrounding environment using a measured point cloud of 360${}^{\circ }$ from the 3D LIDAR and determines the gazing range depending on the unevenness evaluation. Then, the 3D LIDAR rotates at a low speed in the gazing range and a high speed in the remaining range. This controlled motion of the 3D LIDAR results in a measurement with a relatively high spatial resolution in the gazing range. However, the 3D sensing system has a constant measurement range of 360${}^{\circ }$ and a trade-off between the spatial and temporal resolutions. Therefore, the system has difficulty in improving both spatial and temporal resolutions. In addition, the rotation control of LIDAR may not be able to respond to the fast motion of the measured target owing to the latency of the 360${}^{\circ }$ unevenness evaluation.

To overcome the drawbacks of the existing methods, we propose an adaptive 3D sensing system with a direct drive motor. The direct drive motor can adaptively control the angular velocity of the rotation and has back drivability. Therefore, the proposed adaptive 3D sensing system realizes not only a high-speed rotating motion for 360${}^{\circ }$ sensing, such as that in Nagai’s 3D sensing system, but also a high-speed reciprocating motion for local 3D sensing at a high spatio-temporal resolution. Furthermore, the motion of the direct drive motor can be switched with low latency because the proposed system controls the direct drive motor for each frame of the image obtained at a high frame rate. Consequently, the low-latency visual feedback achieves continuous tracking and measurement of fast-moving objects without any tracking algorithms, such as Kalman Filter. 

## 3. Method of AdjustSense

### 3.1. Concept and Configuration

With reference to 3D sensing systems, the spatial resolution *S*, temporal resolution *T*, and measurement range *A* are connected in a trade-off relationship, as expressed in Equation (1):

(1) S × T × A = Const.

For example, the temporal resolution *T* of omnidirectional LIDAR is small because its measurement range *A* is set to 360${}^{\circ }$. In contrast, LIDAR, which has a smaller measurement range has an increased temporal resolution. As another example, the RealSense D435 system obtains depth information, not only with a wide field of view at low speed, but also with a narrow field of view at high speed [17]. In particular, the measurement rate is 30 fps, and the spatial resolution is 1280 × 720 px in the low-speed mode. By focusing the measurement range, the measurement rate is improved to 300 fps, and the spatial resolution decreases to 848 × 100 px in the high-speed mode. However, in these conventional 3D sensing systems, the spatio-temporal resolution and measurement range are defined in advance and are constant during operation. On the other hand, AdjustSense controls the spatio-temporal resolution and the measurement range dynamically during operation, based on the trade-off relationship.

**Figure 2:**
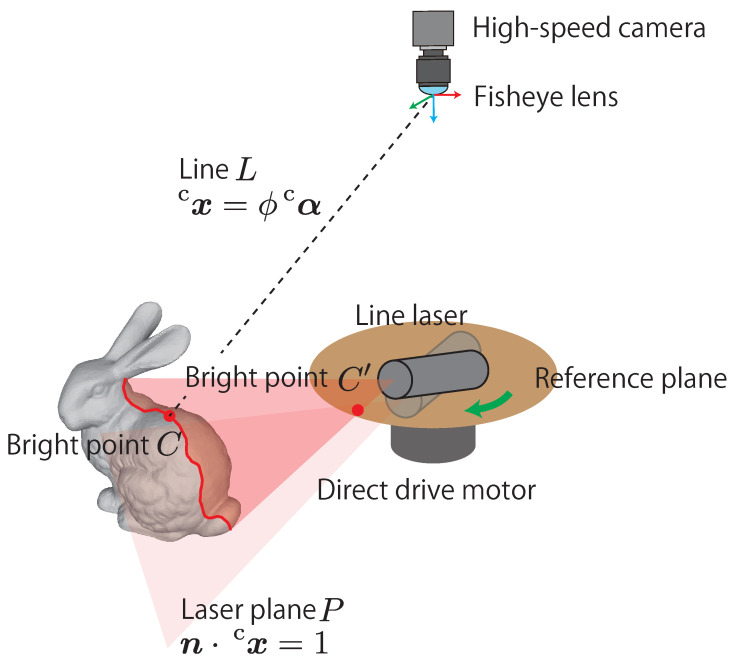
Configuration of AdjustSense.

**Figure 3:**
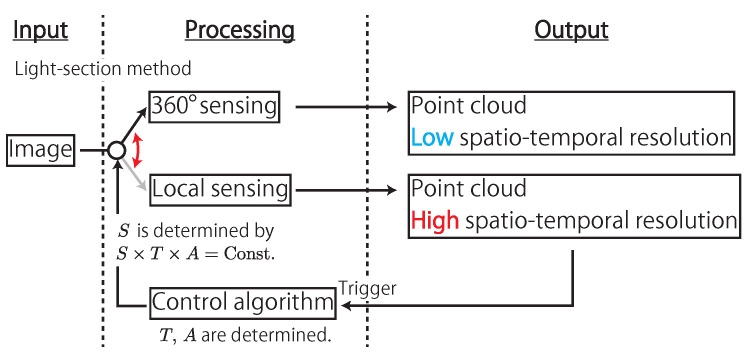
Flowchart of AdjustSense.

AdjustSense comprises a high-speed camera with a fisheye lens, a direct drive motor, and a line laser, as shown in Figure 2. Figure 3 shows the flowchart of AdjustSense. Firstly, the high-speed omnidirectional camera obtains an image in which the line laser is irradiated. Then, with the image, the 3D positions of the bright points of the line laser are calculated using the light-section method [18]. By accumulating the calculated 3D positions on the line laser in chronological order, a wide range of 3D sensing result can be obtained. 

**Figure 4:**
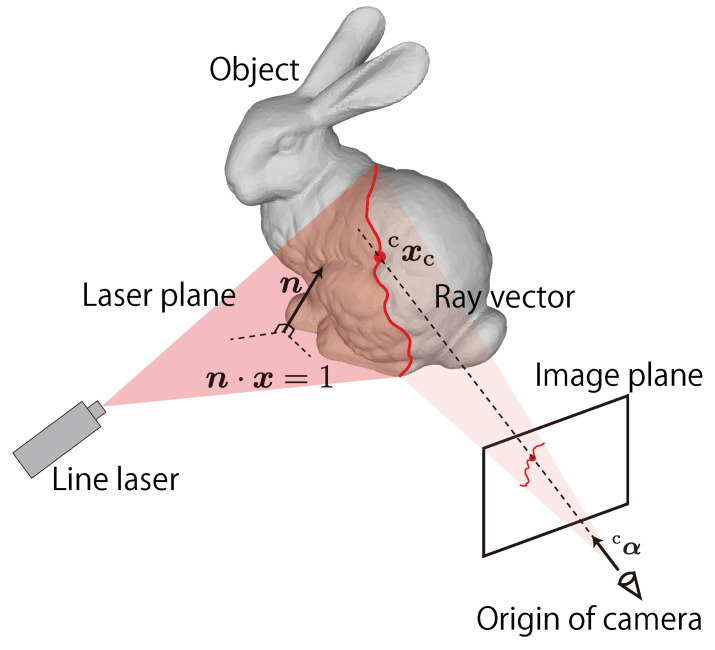
Principle of light-section method.

The principle of light-section method is based on optical triangulation using the ray vector of the camera and the plane of the line laser as shown in Figure 4. The 3D position ${}^{c}x_{c}$ of one of the bright points in Figure 4 is calculated with the geometric relationship of the intersection of the ray vector and the laser plane. The light-section method is suitable for AdjustSense because it can easily control the spatio-temporal resolution S,T and measurement range *A* by changing the direction of the line laser. At this point, AdjustSense defines the measurement range *A* as the angle range of horizontal scanning by using the line laser and the direct drive motor. Therefore, AdjustSense can change the *T* and *A* of the measurement by controlling the horizontal rotation speed and angle of the line laser with the direct drive motor. Then, *S* is also changed based on the trade-off relationship Equation (1). In the system, the point cloud obtained using the light-section method is used as a trigger to adaptively change the rotation speed and angle of the line laser. By integrating not only a high-speed sensor but also a high-speed actuator, a 3D sensing system based on the light-section method that can adaptively adjust the spatio-temporal resolution and the measurement range with low latency can be constructed.

### 3.2. Adaptive 3D Sensing Based on Light-Section Method

**Figure 5:**
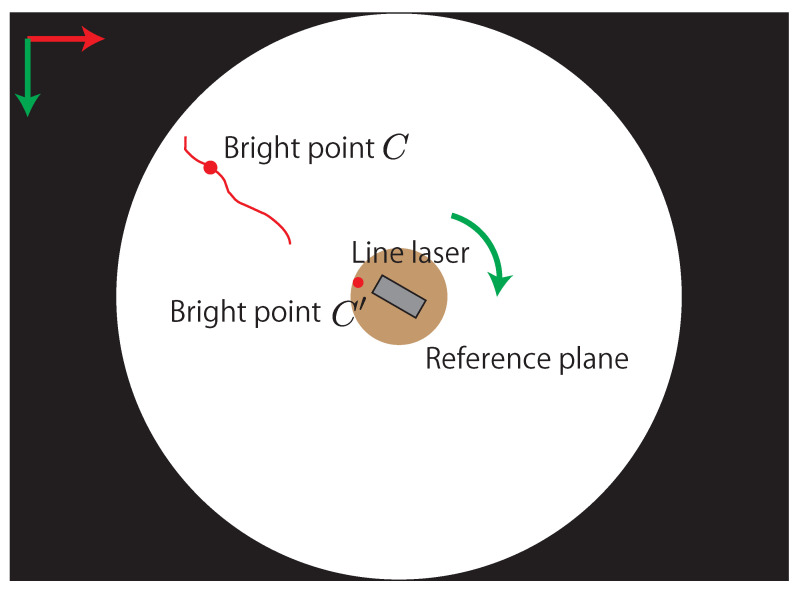
Overview of image obtained using high-speed omnidirectional camera.

In this study, a high-speed camera with a fisheye lens and a line laser is utilized for 3D sensing based on the light-section method. Furthermore, the line laser is attached to the direct drive motor to control the direction of the line laser at a high speed and adaptively change the measurement range. Even when the line laser is irradiated to the entire circumference, the high-speed omnidirectional camera can keep the line laser in the image owing to its wide field of view. Figure 5 shows an overview of the image obtained with the high-speed omnidirectional camera for the light-section method. In the figure, the rotating line laser attached to the direct drive motor appears in the center, and the bright line of the line laser appears around the line laser. At a certain point, the 3D position ${}^{c}x_{c}$ of one of the bright points *C* can be calculated using the geometric relationship between the image coordinate ${{(}^{i}u_{c},{\;}^{i}v_{c})}^{T}$ of the bright point *C* and the plane equation n·x = 1 of the line laser, as shown in Figure 4 and Equation (2),

(2) $$\begin{array}{c} \left\{\begin{array}{cc} {}^{c}x_{c} & \;=\;\phi {\;}^{c}\alpha \\ n\cdot {\;}^{c}x_{c} & \;=\;1. \end{array}\right. \end{array}$$

Here, the first equation of Equation (2) denotes the line *L* connecting the origin of the camera coordinate to the bright point *C*, as shown in Figure 4. The direction vector ${}^{c}\alpha$ of the line *L* can be calculated from the image coordinate ${{(}^{i}u_{c},{\;}^{i}v_{c})}^{T}$ of the bright point *C*  based on Scaramuzza’s fisheye model [19] as in Equation (3).

(3) $${}^{c}\alpha \;=\;\left(\begin{array}{c} u_{id}\\ v_{id}\\ a_{0}+a_{2}\rho^{2}+a_{3}\rho^{3}+a_{4}\rho^{4} \end{array}\right),$$

where $u_{id}$ and $v_{id}$ are ideal image coordinates of the bright point *C*, $a_{0}$, $a_{2}$, $a_{3}$ and $a_{4}$ are Scaramuzza’s parameters, and ρ is a distance from a distortion center $\rho =\sqrt{u_{id}^{2}+v_{id}^{2}}$. The variable ϕ means a scale factor of ${}^{c}\alpha$ to match the distance of the 3D position ${}^{c}x_{c}$. The light-section method in AdjustSense requires fast and accurate extraction of all the image coordinates of the bright points in the images obtained from the high-speed omnidirectional camera. The second equation in Equation (2) denotes the laser plane *P* designed to obtain 3D points using disparity between the line laser and the camera. In AdjustSense, the geometric relationship between the high-speed omnidirectional camera and the line laser changes dynamically because the line laser is adaptively rotated by the direct drive motor at high speed. Therefore, AdjustSense must be calibrated such that the normal vector n of the plane of the line laser can be always updated.

**Figure 6:**
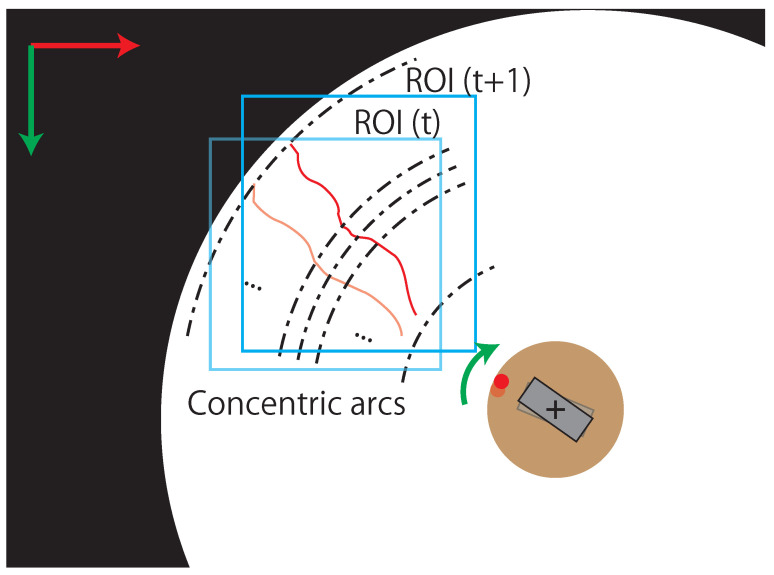
Concentric arcs and ROIs for fast and accurate extraction of bright points.

To obtain accurate image coordinates of the bright points, the luminance center of gravity (CoG) is calculated in the light-section method. In particular, the luminance CoGs are calculated in concentric arcs because the line laser is irradiated radially from the center of the image, as shown in Figure 6. This process not only reduces unnecessary calculations for 3D sensing, but also obtains an accurate position of the bright points from the line laser. Furthermore, when all luminance CoGs are detected by the process, a region of interest (ROI) is set up with a margin to enclose all luminance CoGs, as shown in Figure 6. By reducing the processing area within the ROI, the amount of computation required to extract the luminance CoGs can be reduced. In addition, the position variation of the line laser in the image is small because the image of the line laser is captured by the high-speed omnidirectional camera. Therefore, updating the ROI with a margin from the luminance CoGs allows the line laser to remain inside the ROI.

To calibrate the normal vector n of the line laser, the image coordinate ${{(}^{i}u_{c^{\prime }},{\;}^{i}v_{c^{\prime }})}^{T}$ of the bright point $C^{\prime }$, which is shown in Figure 2, is utilized. The bright point $C^{\prime }$ is on a circular reference plane fixed to the direct drive motor. When the position of the line laser is defined such that a part of the line laser is irradiated on the fixed reference plane, the image coordinate of $C^{\prime }$ changes dynamically depending on the rotation of the direct drive motor. The correspondence relationship between the image coordinate of $C^{\prime }$ and the normal vector n of the line laser is utilized by a polynomial-fitting-based calibration [20]. In the calibration, the intrinsic parameter of the omnidirectional camera and a normal vector n of the line laser are calculated using Zhang’s method [21]. Simultaneously, the image coordinate of the bright point $C^{\prime }$ on the reference plane is calculated. By changing the direction of the line laser and repeating the process, multiple correspondences between the normal vectors n of the laser plane and the image coordinates of the bright point $C^{\prime }$ can be obtained. Finally, these correspondences are regressed into a polynomial function $n=f{(}^{i}u_{c^{\prime }},{\;}^{i}v_{c^{\prime }})$. Consequently, the normal vector n of the line laser can be calculated at any given moment by using the image and the polynomial function.

### 3.3. Control Algorithm for Adjustable Spatio-Temporal Resolution and Measurement Range

**Figure 7:**
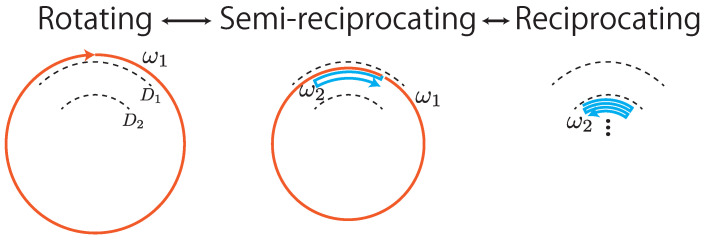
Three control modes of direct drive motor. Angular velocities during rotation and reciprocation are $\omega_{1}$ and $\omega_{2}$, respectively.

A direct drive motor is a gearless servo motor with high torque. It is capable of high-precision positioning and is designed to be driven without a reduction mechanism. The gearless nature of a direct drive motor allows for a high-speed back drive. Therefore, the direct drive motor is considered suitable for devices that realize both rotational and high-speed reciprocating motion, as in AdjustSense. Figure 7 shows an overview of three control modes of the direct drive motor for adjustable spatio-temporal resolution and measurement range. Although the indicator for switching the three control modes can be arbitrarily determined, in this research it is based on the distance of the measured points from the high-speed omnidirectional camera to sense the surrounding environment and detect nearby objects in detail. The first control mode is the “rotating mode” for 360${}^{\circ }$ 3D sensing at high speed. When an object approaches the 3D sensing system, the control mode switches to one rotating and several reciprocating motions for 360${}^{\circ }$ and local 3D sensing. This is referred to as the “semi-reciprocating mode” in this study. In this mode, at the expense of measurement time, it is possible to not only measure the 360${}^{\circ }$ environment, but also focus on the local area around the object. When the object approaches, the line laser is reciprocated only around the object at a high speed for local 3D sensing at a high spatio-temporal resolution; this is called the “reciprocating mode.”

**Figure 8:**
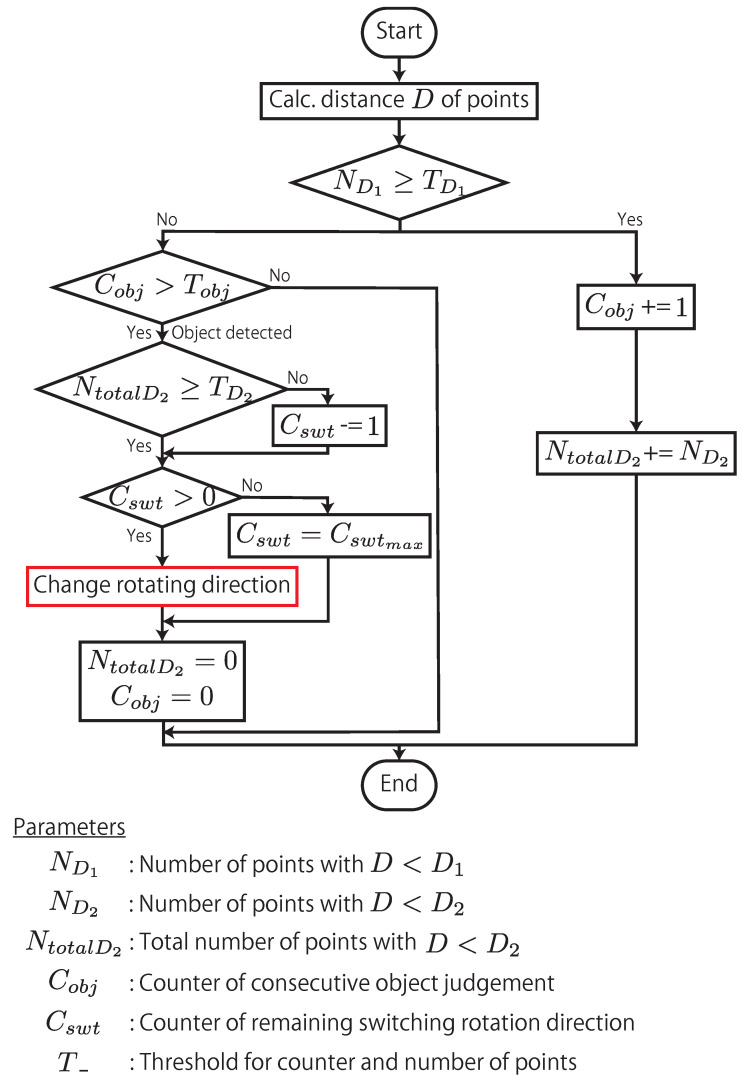
Flowchart of control algorithm for direct drive motor.

To adaptively switch between these three control modes, AdjustSense controls the timing and the number of remaining counts for switching the rotating direction according to the flowchart shown in Figure 8. Firstly, the distances of the measured 3D points from one image captured by the camera are calculated. When the number of points $N_{D_{1}}$, whose distance is less than the value $D_{1}$, is greater than the threshold $T_{D_{1}}$, the object to be scanned is regarded as a candidate of the measurement target, and the counter of consecutive object judgment $C_{obj}$ is increased. Simultaneously, the number of points $N_{D_{2}}$, whose distance is less than $D_{2}\;\left(<D_{1}\right)$, is added to the total number of measured 3D points $N_{totalD_{2}}$. When the line laser is kept irradiating on the object, the increment of the counter is repeated. However, when the laser scanning reaches the edge of the object, the number of points $N_{D_{1}}$ is less than $T_{D_{1}}$, and the continuous judgment is completed. When this occurs, if the counter $C_{obj}$ is more than $T_{obj}$, the scanned object is detected as a measurement target. After the object detection, if the total number $N_{totalD_{2}}$ is less than the threshold $T_{D2}$, the counter $C_{swt}$ of remaining switching rotation direction is reduced. If the counter $C_{swt}$ is greater than zero, the rotating direction is changed, and the reciprocating motion of the line laser around the measurement target is repeated; if it is zero, the reciprocating motion is terminated and, AdjustSense returns to the rotating motion for 360${}^{\circ }$ 3D sensing. By repeating the above processes, it is possible to adaptively switch between 360${}^{\circ }$ and local 3D sensing.

## 4. Experiments

### 4.1. Experimental Configuration

**Figure 9:**
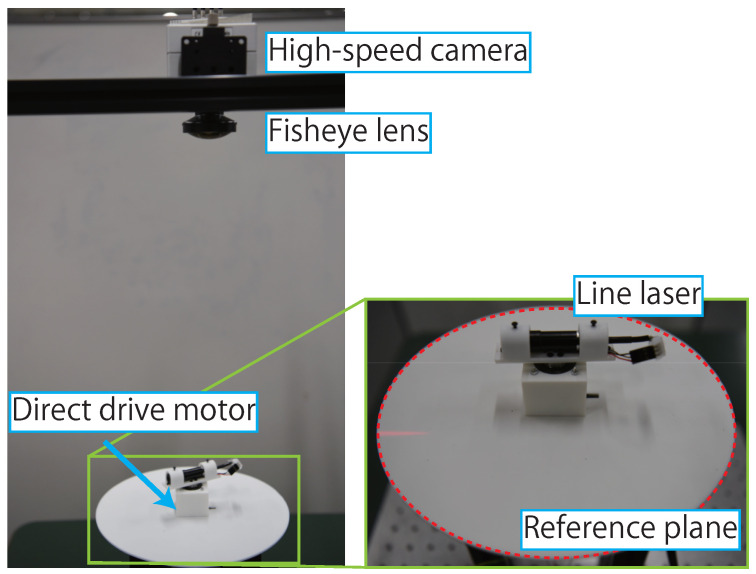
Experimental configuration of adaptive 3D sensing system.

To evaluate the performance of AdjustSense, we built an experimental configuration as shown in Figure 9. The experimental configuration compromised a high-speed camera (Optronics, CP70-2-C-1000 @1000 fps) with a fisheye lens (Fujinon, FE185C086HA-1), a line laser (Kikoh Giken, MLXL-D13-660-120), and a direct drive motor (MTL, MDH-3018-108KE). We introduced a microcomputer (mbed NXP, LPC1768) to send the control signal to the motor driver (MTL, MC-200-7220A) of the direct drive motor. A PC (Intel Core i9-9900K @3.60 GHz) was used to process the images for 3D sensing using the light-section method and to send triggers for switching the rotating direction of the direct drive motor. The processing rate of 1000 fps for light-section method and control trigger transmission was achieved. Consequently, AdjustSense realized low-latency visual feedback of 1 ms from image acquisition to control trigger transmission. The high-speed omnidirectional camera was mounted such that the Z-axis of the camera was parallel to the direction of the gravitational force. Therefore, the distances from the high-speed camera to the measured 3D points calculated in the control algorithm for the direct drive motor were on the XY plane of the camera coordinates in the experimental configuration.

To determine the motion of the direct drive motor, we set the parameters shown in Section 3.3. Firstly, the rotation speeds $\omega_{1}$ and $\omega_{2}$ of the direct drive motor for rotating and reciprocating motions were set at 500rpm and 200rpm, respectively. Then, as parameters for switching the control modes of the direct drive motor, the distances $D_{1}$ and $D_{2}$ from the high-speed camera to the measured 3D points on the XY plane were set as 1.5m and 1.0m, respectively. The thresholds $T_{D1}$ and $T_{D2}$ were set to 20 and 10, respectively. Furthermore, the threshold $T_{obj}$ for detecting the measurement target was set to 5, and the maximum value $C_{swt_{max}}$ of the counter $C_{swt}$ was set to 3.

### 4.2. Experimental Details

**Figure 10:**
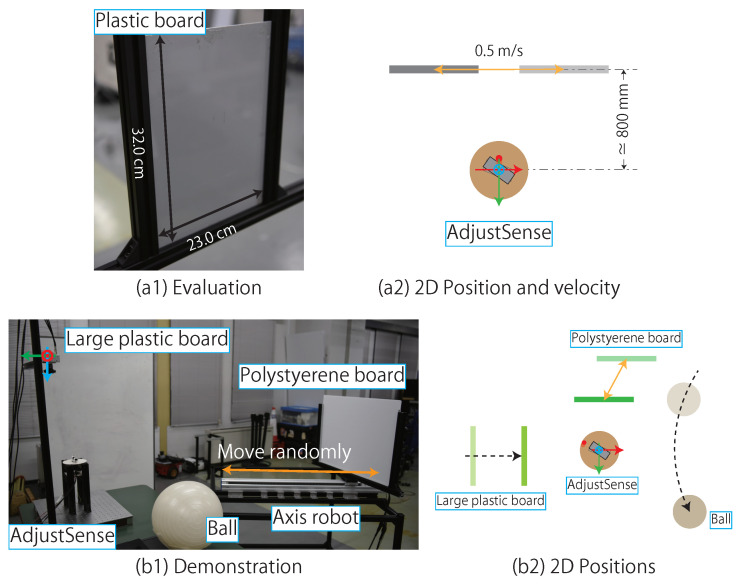
Measured objects for (**a1**) evaluating spatio-temporal resolution, measurement range and measurement accuracy, (**b1**) demonstrating 360${}^{\circ }$, and local sensing. (**a2**) represents 2D position and velocity of measured object in (**a1**) evaluation. (**b2**) represents 2D positions of measured objects in (**b1**) demonstration.

Two experiments were conducted in this study. In the first experiment, we quantitatively evaluated the spatio-temporal resolution, the measurement range, and the measurement accuracy in three control modes of the direct drive motor. In the evaluation, the point cloud from AdjustSense was compared with that of a LIDAR (Velodyne, HDL-32E) introduced as a baseline. The measured object was a plastic board as shown in Figure 10a. In the experimental procedure, the position of the measured object was first fixed approximately 800 mm from the Z-axis of the camera. Next, we manually changed the values of $D_{1}$ and $D_{2}$ when measuring with AdjustSense, and measured object in each of three control modes. The same object was also measured using LIDAR. The spatio-temporal resolution and measurement range were calculated using the measured point cloud around the measured object and the motion logs in each system. Furthermore, by taking advantage of the fact that the measured object was planar, a planar fitting was performed on the obtained point cloud, and the distance between the plane and the measured point was calculated as the measurement error, and the standard deviation of the measurement error was evaluated as the measurement accuracy. The above experimental process was performed for the same measured object in a moving situation. The measured object was attached to an axis robot (MISUMI RSH220B-C21A-N-F1-5-700). The direction of the axis robot was installed parallel to the X-axis of the camera and the velocity was fixed at 0.5 m/s. 

In the second experiment, we demonstrated the 360${}^{\circ }$ and local 3D sensing with the following experimental configuration. The measured objects were a polystyrene board attached to the axis robot, a large plastic board, and a ball attached to the bar, as shown in Figure 10(b1). The measured objects were located around the constructed 3D sensing system according to Figure 10(b2). In the demonstration, the polystyrene board was randomly moved by controlling the speed and position of the axis robot. The large board and ball were manually moved. We evaluated whether the control mode of the direct drive motor was adaptively changed with low latency according to the positions of the measured objects even if the positions were changed at high speeds. In the demonstration, we confirmed not only the detection of the approaching object during the 360${}^{\circ }$ 3D sensing, but also the tracking of a nearby object at a high spatio-temporal resolution in accordance with the relative position of the object.

## 5. Results

### 5.1. Evaluation of Spatio-Temporal Resolution, Measurement Range and Measurement Accuracy

**Figure 11:**
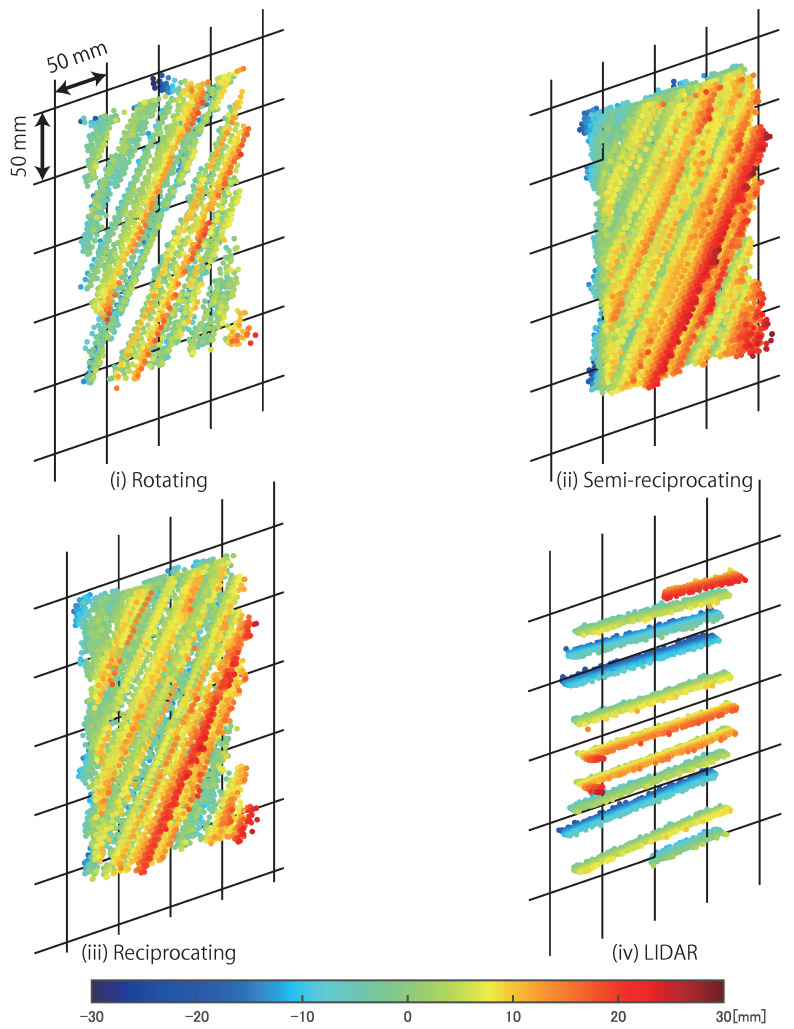
Measured 3D points on fixed plastic board in three control modes of AdjustSense and LIDAR, which were obtained from 20 updates of 3D measurement. Color bar indicates distance defined as measurement error which is from output plane when point cloud is applied to plane fitting.

Figure 11 shows measurement results in the three control modes of AdjustSense and LIDAR. Table 1 presents the results of the spatio-temporal resolution, the measurement range, and the measurement accuracy calculated from the measured 3D points shown in Figure 11. The spatial resolution indicates the number of measured 3D points in 1 ${\mathrm{cm}}^{2}$ around the plastic board during one update of 3D sensing. The temporal resolution indicates the number of times the entire measurement range is updated per second, and the measurement range indicates the angle range of horizontal scanning until the 3D sensing is updated. The figure shows that the LIDAR had a smaller spatial resolution perpendicular to the direction of rotation compared with that in the direction of rotation. On the other hand, AdjustSense captured images of the line laser scanned at high speed by the camera, and thus was able to obtain a point cloud of the entire measured object. Overall, AdjustSense based on optical triangulation in the light-section method had better measurement accuracy than LIDAR based on the time of flight methodology at a distance of approximately 800 mm in the table.

By comparing the three control modes of the direct drive motor, it was confirmed that AdjustSense could control the spatio-temporal resolution S,T and measurement range *A* according to the trade-off relationship Equation (1). In the rotating mode, the measurement range was almost 360${}^{\circ }$ because the line laser continued rotating; however, the spatio-temporal resolution was smaller than that of LIDAR. The spatial resolution difference was due to the large update frequency of the time of flight of LIDAR compared to the 1000-Hz update frequency in the light-section method of AdjustSense, and the temporal resolution difference was due to the difference in the angular velocity between the direct drive motor of AdjustSense and LIDAR. Therefore, the spatio-temporal resolution could be improved by increasing not only the frame rate of the camera but also the angular velocity of the motor. Meanwhile, compared to the measurement result in the rotating mode, the spatial resolution in the semi-reciprocating mode was high. However, the temporal resolution in this mode was low because the measurement range was larger than that in the rotating mode. The value of S×T×A in the semi-reciprocating mode was larger than that in the rotating mode because the semi-reciprocating mode consisted of one motion in the rotating mode and three motions in the reciprocating mode. In fact, as shown in Table 2, the respective values of S,T, and *A* in the semi-reciprocating mode were almost equal to the sum of the values in the rotating mode and three times the value in the reciprocating mode. To improve the spatial and temporal resolutions simultaneously, AdjustSense concentrated the measurement range from almost 360${}^{\circ }$ in the rotating mode to the periphery of the measured object in the reciprocating mode, resulting in exceeding the spatio-temporal resolution of LIDAR. In the switch of the control mode, the value of S×T×A in the reciprocating mode was almost equal to that in the rotating mode. This indicated that AdjustSense could distribute the spatio-temporal resolution and measurement range according to the trade-off relationship Equation (1) and the predefined parameters of the direct drive motor.

**Figure 12:**
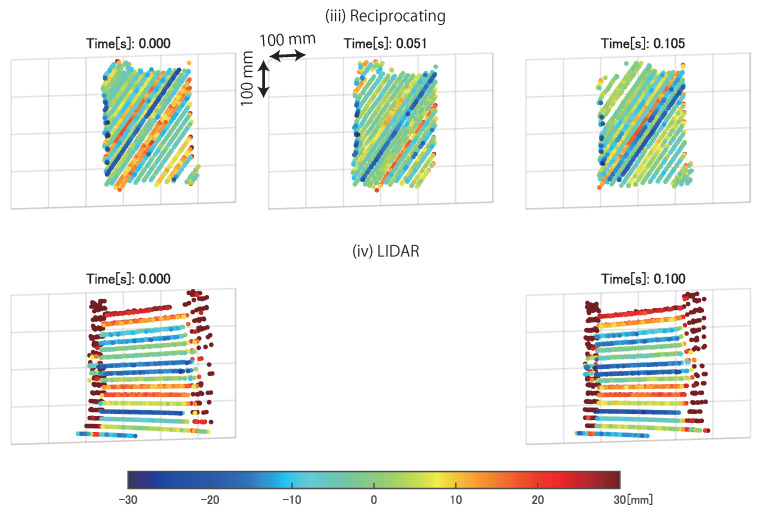
Measured 3D points on moving plastic board in reciprocating mode of AdjustSense and LIDAR. Each figure was obtained from one update of 3D measurement. Color bar indicates distance defined as measurement error which is from output plane when point cloud is applied to plane fitting.

Figure 12 shows the time transition of measurement points on the moving plastic board in the reciprocating mode of AdjustSense and LIDAR. Table 3 presents the spatio-temporal resolution, measurement range, and measurement accuracy calculated from the results in Figure 12. As in the experiment with the fixed plastic board, AdjustSense exceeded the spatio-temporal resolution of LIDAR by focusing the measurement range. Therefore, AdjustSense could recognize the fluctuation and shape of the measured object in more detail. In addition, AdjustSense controlled the direction of the line laser at high speed to continue tracking the moving object without losing sight of it. 

### 5.2. Demonstration of 360${}^{\circ }$ and Local 3D Sensing in AdjustSense

**Figure 13:**
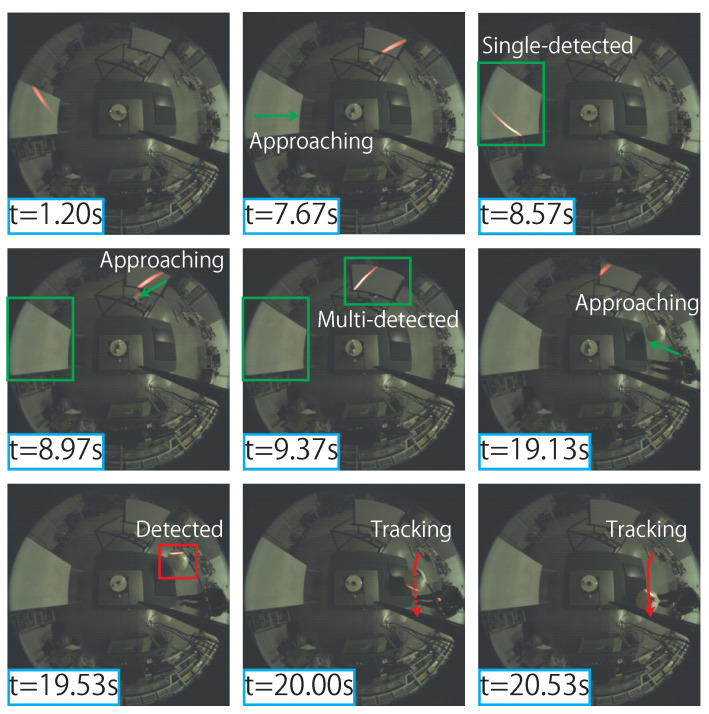
Demonstration of AdjustSense.

Figure 13 shows the behavior of AdjustSense when the positions of the measured objects in the surroundings were changed. As shown in the figure, AdjustSense measured the entire circumference by rotating the line laser at high speed (t=1.20s). In this timeframe, the measured 3D points with distances less than $D_{1}$ did not exist; the rotation speed was 500 rpm, and the rotating direction of the line laser was maintained constant. When the rotating mode of the direct drive motor was activated during the experiment, the large plastic board approached the 3D sensing system from the left side in the obtained image (t = 7.67s). The plastic board was then judged as a measurement target in the control algorithm because the number of measured 3D points on the plastic board whose distances were less than $D_{1}$ was greater than $T_{D_{1}}$. Therefore, the rotating direction was changed at the edge of the plastic board, and the control modes of the direct drive motor were switched from rotating mode to semi-reciprocating mode (t = 8.57s). Subsequently, the position of the plastic board was fixed, and the line laser reciprocated around the plastic board during one rotation. Moreover, the polystyrene board also approached the 3D sensing system from the top of the image obtained by the axis robot (t = 8.97s). When the polystyrene board was judged as a measurement target in the control algorithm (t = 9.37s), the line laser reciprocated around not only the plastic board, but also the polystyrene board during one rotation in the semi-reciprocating mode. Subsequently, the position of the polystyrene board fluctuated under the control of the axis robot. Therefore, AdjustSense switched to the semi-reciprocating mode, in which the line laser reciprocated only around the plastic board when the polystyrene board moved away and around both the plastic board and the polystyrene board when the polystyrene board moved closer. In addition, when the ball approached the 3D sensing system from the right side in the obtained image during the semi-reciprocating mode (t = 19.13s), the distances of the measured 3D points on the ball were less than $D_{2}$, and the control mode of the direct drive motor was switched from the semi-reciprocating mode to the high-speed reciprocating mode, whose measurement range was focused on the area around the ball. Consequently, the measurement range tracked the ball even when the position of the ball changed randomly at high speed until the distances of the measured 3D points on the ball were greater than $D_{2}$ (t = 19.53–20.53 s).

**Figure 14:**
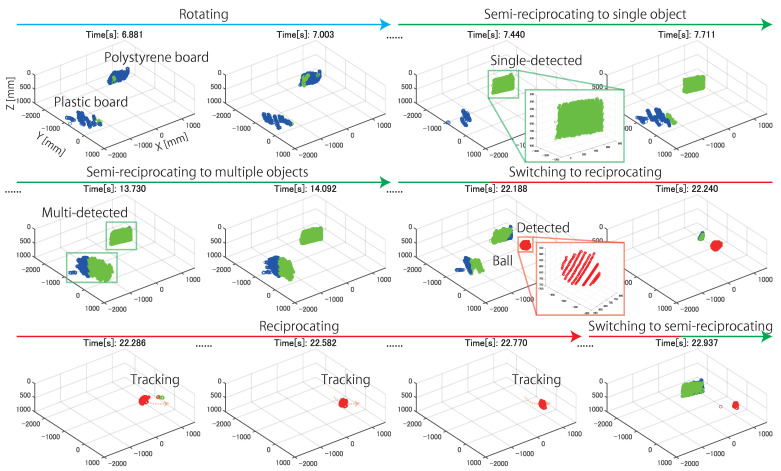
Time transition of 3D sensing results from AdjustSense. Blue, green, and red dots indicate measured 3D points whose distances from high-speed omnidirectional camera were more than $D_{1}$, less than $D_{1}$, and less than $D_{2}$, respectively.

Figure 14 shows the measured 3D points in the experiment. These 3D sensing results are the outputs of the 3D points during one update for each control mode of the direct drive motor. The times on these figures represent the time since the start of the experiment. In the figure, it is confirmed that sparse 3D points were obtained for the entire circumference when the line laser was rotated at high speed in the rotating mode. When the object approached the 3D sensing system, compared to the measurement result in the rotating mode, the spatial resolution of the measured 3D points around the object was improved by rotating and reciprocating the line laser in the semi-reciprocating mode. However, the update time of the semi-reciprocating mode increased because the measurement range was increased. In the semi-reciprocating mode, not only a single object but also multiple objects could be detected and were measured at a high spatial resolution at the expense of the update time by combining the rotational and reciprocating motions. In addition, when the object approached to the 3D sensing system, the control mode was switched to the reciprocating mode to measure only that object at a high spatio-temporal resolution. Compared to the measurement result for the rotating mode, the spatial and temporal resolutions of the object were improved by focusing the measurement range from 360${}^{\circ }$ to the local area around the object. The high spatio-temporal resolution of the measurement enabled tracking of the measurement target without the need to estimate the fast positional variations of the measurement targets using any tracking algorithms. AdjustSense used the thresholds shown in the flowchart of Figure 8 to detect the decrease in the number of 3D points measured when the beam of the line laser reached the edge of the measurement target. After detection, AdjustSense switched the rotational direction of the line laser with low latency of 1 ms in the reciprocating mode to change the measurement range at high speed as in tracking.   Although AdjustSense can improve the spatio-temporal resolution by focusing the measurement range, the focused measurement range restricts the detection of another object approaching from the outside of the measurement range until the measured object in the reciprocating mode moves away and the AdjustSense switches the control mode. That is the limitation of AdjustSense based on the trade-off relationship between the spatio-temporal resolution and the measurement range in Equation (1). Through this experiment, we confirmed that the following operations could be adaptively switched with low-latency visual feedback in a single 3D sensing system: 360${}^{\circ }$ 3D sensing by high-speed rotation of the line laser, 3D sensing at a high spatial resolution by reciprocating the line laser around multiple approaching objects during one rotation, and 3D sensing and tracking at a high spatio-temporal resolution by focusing the measurement range only on a single nearby object.

We consider an UAV system for navigation and safe landing on unstable ground as one potential application of AdjustSense [22]. The 360${}^{\circ }$ 3D sensing result from the rotating mode of AdjustSense is effective for avoiding surrounding obstacles and moving objects during flight. Moreover, the focused 3D sensing at high spatio-temporal resolution in the reciprocating mode of AdjustSense can be applied to obtain the 3D shape of the landing point. AdjustSense can switch between the measurement task depending on the motion sequence of the UAV without the need for the installation of multiple 3D sensors. 

## 6. Conclusions

In this study, we proposed AdjustSense, which can switch between multiple measurement tasks with low latency. By calculating the 3D positions using images obtained from the high-speed camera and controlling the timing and number of remaining counts of changing the rotating direction of the line laser with the measured 3D points, AdjustSense adaptively switched the three control modes of the direct drive motor with low latency of 1 ms. Through our experiments, we demonstrated that AdjustSense realizes multiple tasks in 3D sensing: 360${}^{\circ }$ 3D sensing by rotating the line laser at high speed, 3D sensing at a high spatial resolution by reciprocating the line laser around multiple approaching objects, and 3D sensing and tracking at a high spatio-temporal resolution by focusing the measurement range. In addition, we evaluated the adaptive adjustment of the spatio-temporal resolution and the measurement range with the measurement results in three control modes.

Future works include detecting objects outside the focused measurement range in the reciprocating mode and optimizing the control behavior of the direct drive motor by adaptively varying the threshold parameters set for the motion determination based on not only the 3D sensing result, but also the image recognition [23,24] of the measured ambient environment. This optimization can efficiently distribute the spatio-temporal resolution and measurement range according to the trade-off relationship Equation (1), depending on the content of the measured object and its position.

## Figures and Tables

**Table 1:** Spatio-temporal resolution, measurement range, and measurement accuracy in three control modes of AdjustSense and LIDAR calculated with 3D point obtained from fixed plastic board.

	AdjustSense			Baseline
	(i) Rotating	(ii) Semi-Reciprocating	(iii) Reciprocating	(iv) LIDAR
*S*: Spatial resolution [$\mathrm{points}/({\mathrm{cm}}^{2}\cdot \mathrm{update})$]	0.789	6.997	2.039	1.559
*T*: Temporal resolution [update/s]	8.097	3.959	19.57	10.00
*A*: Measurement range [deg/update]	359.9	539.1	55.35	360.0
Measurement accuracy [mm]	6.741	6.662	6.103	9.746
S×T×A	2299.2	14,934	2208.6	–

**Table 2:** Relationship between three control modes in terms of spatio-temporal resolution and measurement range derived from evaluation experiment results.

	(ii)	(i) × 1 + (iii) × 3
*S*: Spatial resolution [$\mathrm{points}/({\mathrm{cm}}^{2}\cdot \mathrm{update})$]	6.997	6.906
*T*: Temporal resolution [update/s]	3.959	3.612
*A*: Measurement range [deg/update]	539.1	525.95

**Table 3:** Spatio-temporal resolution, measurement range, and measurement accuracy in reciprocating mode of AdjustSense and LIDAR calculated with 3D point obtained from moving plastic board.

	AdjustSense (iii) Reciprocating	Baseline (iv) LIDAR
*S*: Spatial resolution [$\mathrm{points}/({\mathrm{cm}}^{2}\cdot \mathrm{update})$]	1.859	1.525
*T*: Temporal resolution [update/s]	19.05	10.00
*A*: Measurement range [deg/update]	44.33	360.0
Measurement accuracy [mm]	8.965	12.67

## References

[1] Mancini F., Dubbini M., Gattelli M., Stecchi F., Fabbri S., Gabbianelli G. (2013). Using Unmanned Aerial Vehicles (UAV) for High-Resolution Reconstruction of Topography: The Structure from Motion Approach on Coastal Environments. Remote Sens..

[2] Ishikawa R., Roxas M., Sato Y., Oishi T., Masuda T., Ikeuchi K. A 3D Reconstruction with High Density and Accuracy Using Laser Profiler and Camera Fusion System on a Rover. Proceedings of 2016 Fourth International Conference on 3D Vision (3DV).

[3] Aoyama T., Li L., Jiang M., Inoue K., Takaki T., Ishii I., Yang H., Umemoto C., Matsuda H., Chikaraishi M. (2018). Vibration Sensing of a Bridge Model Using a Multithread Active Vision System. IEEE/ASME Trans. Mechatron..

[4] Zhang C., Xu J., Xi N., Jia Y., Li W. Development of an Omni-directional 3D Camera for Robot Navigation. Proceedings of IEEE/ASME International Conference on Advanced Intelligent Mechatronics (AIM).

[5] Higuchi H., Fujii H., Taniguchi A., Watanabe M., Yamashita A., Asama H. (2019). 3D Measurement of Large Structure by Multiple Cameras and a Ring Laser. J. Robot. Mechatron..

[6] Barnard S.T., Fischler M.A. (1982). Computational Stereo. ACM Comput. Surv..

[7] Massa J.S., Buller G.S., Walker A.C., Cova S., Umasuthan M., Wallace A.M. (1998). Time-of-flight optical ranging system based on time-correlated single-photon counting. Appl. Opt..

[8] Payeur P., Desjardins D., Kamel M., Campilho A. (2009). Structured Light Stereoscopic Imaging with Dynamic Pseudo-random Patterns. Image Analysis and Recognition.

[9] Keselman L., Woodfill J., Grunnet-Jepsen A., Bhowmik A. Intel RealSense Stereoscopic Depth Cameras. Proceedings of Computer Vision and Pattern Recognition (CVPR).

[10] Zhang Z. (2012). Microsoft Kinect Sensor and Its Effect. IEEE Multimed..

[11] Watanabe Y., Komuro T., Ishikawa M. 955-fps Real-time Shape Measurement of a Moving/deforming Object Using High-speed Vision for Numerous-point Analysis. Proceedings of IEEE International Conference on Robotics and Automation (ICRA).

[12] Namiki A., Shimada K., Ishii Y.K. (2019). Development of an Active High-speed 3-D Vision System. Sensors.

[13] Miyashita L., Kimura Y., Tabata S., Ishikawa M. (2021). High-speed simultaneous measurement of depth and normal for real-time 3D reconstruction. Applications of Digital Image Processing XLIV.

[14] Tabata S., Noguchi S., Watanabe Y., Ishikawa M. High-speed 3D Sensing with Three-view Geometry Using a Segmented Pattern. Proceedings of the IEEE/RSJ International Conference Intelligent Robots Systems (IROS).

[15] Chen J., Gu Q., Gao H., Aoyama T., Takaki T., Ishii I. Fast 3-D shape measurement using blink-dot projection. Proceedings of the IEEE International Conference on Intelligent Robots and Systems. IEEE.

[16] Nagai Y., Kuroda Y. Control of Spatial Points Density According to Object Shape with 3D LiDAR. Proceedings of the SICE 20th System Integration Division (SI2019).

[17] Sonoda T., Sweetser J.N., Khuong T., Brook S., Grunnet-Jepsen A. High-Speed Capture Mode of Intel RealSense Depth Camera D435. https://dev.intelrealsense.com/docs/high-speed-capture-mode-of-intel-realsense-depth-camera-d435.

[18] Curless B., Levoy M. Better Optical Triangulation through Spacetime Analysis. Proceedings of the IEEE International Conference on Computer Vision (ICCV).

[19] Scaramuzza D., Martinelli A., Siegwart R. A Toolbox for Easily Calibrating Omnidirectional Cameras. Proceedings of the IEEE/RSJ International Conference on Intelligent Robots and Systems.

[20] Ikura M., Pathak S., Yamashita A., Asama H. (2021). Polynomial-fitting based calibration for an active 3D sensing system using dynamic light section method. Proceedings of the SPIE.

[21] Zhang Z. (2000). A Flexible New Technique for Camera Calibration. IEEE Trans. Pattern Anal. Mach. Intell..

[22] Ikura M., Miyashita L., Ishikawa M. (2021). Stabilization System for UAV Landing on Rough Ground by Adaptive 3D Sensing and High-Speed Landing Gear Adjustment. J. Robot. Mechatron..

[23] Redmon J., Divvala S., Girshick R., Farhadi A. You only look once: Unified, real-time object detection. Proceedings of IEEE Conference Computer Vision Pattern Recognition (CVPR).

[24] Zhao Q., Sheng T., Wang Y., Tang Z., Chen Y., Cai L., Ling H. M2det: A single-shot object detector based on multi-level feature pyramid network. Proceedings of AAAI Conference on Artificial Intelligence (AAAI2019).

